# Supplementary material for: Optimizing Antimicrobial Efficacy: Investigating the Impact of Zinc Oxide Nanoparticle Shape and Size
Source: Nanomaterials (Basel). 2024 Apr 6;14(7):638. doi: 10.3390/nano14070638 (PMC11013415; doi:10.3390/nano14070638)
Supplement: Supplementary file 1 [file nanomaterials-14-00638-s001.zip › nanomaterials-2940677-supplementary.pdf]

Supplementary Materials

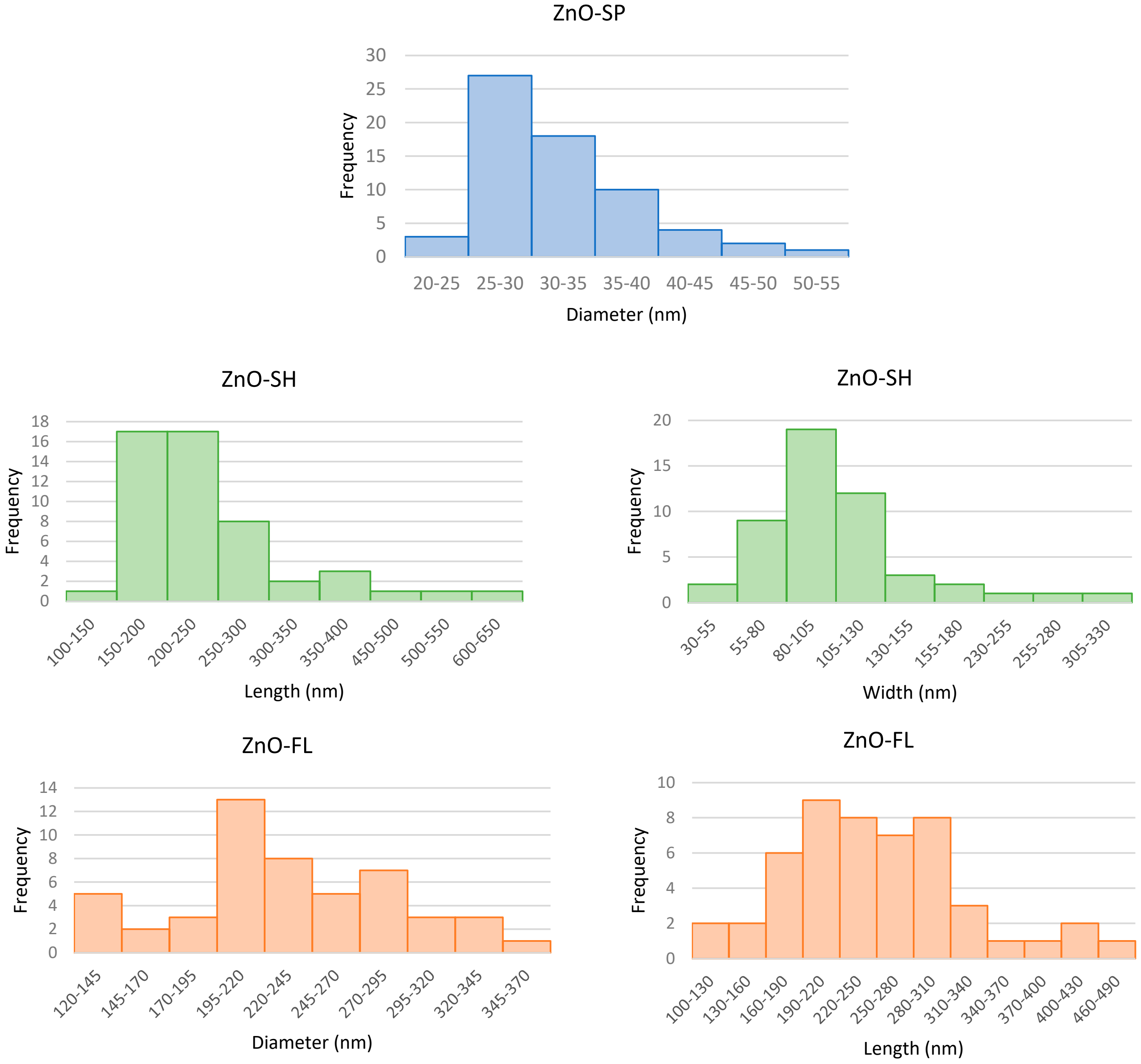

Figure S1. Size distribution of ZnO-SP, ZnO-SH, ZnO-FL.

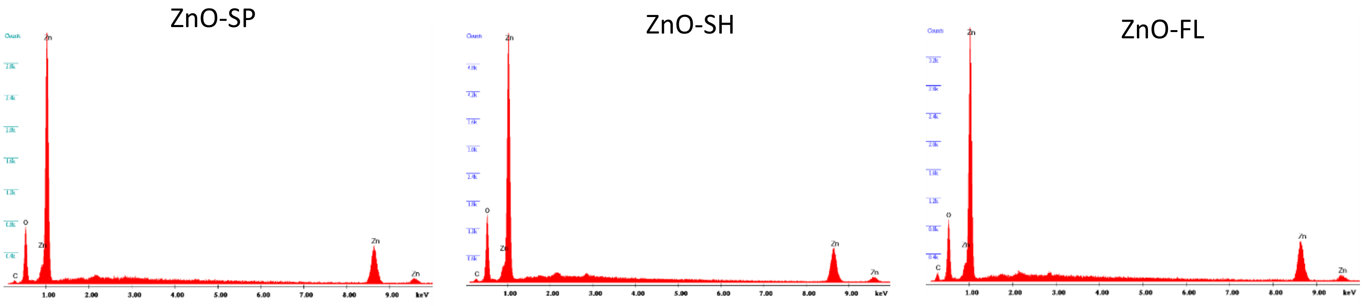

Figure S2. EDS spectra of ZnO-SP, ZnO-SH, ZnO-FL.
